# Genome-wide analysis of the CML gene family and its response to melatonin in common bean (*Phaseolus* *vulgaris* L.)

**DOI:** 10.1038/s41598-023-28445-y

**Published:** 2023-01-21

**Authors:** Hongyan Zhao, Yamei Gao, Yanli Du, Jidao Du, Yiqiang Han

**Affiliations:** 1 College of Life Science and Biotechnology, Heilongjiang Bayi Agricultural University, Daqing, 163319 Heilongjiang People’s Republic of China; 2 National Coarse Cereals Engineering Research Center, Daqing, 163319 Heilongjiang People’s Republic of China; 3 Heilongjiang Provincial Key Laboratory of Environmental Microbiology and Recycling of Argo-Waste in the Cold Region, Heilongjiang Bayi Agricultural University, Daqing, 163319 People’s Republic of China; 4 College of Agriculture, Heilongjiang Bayi Agricultural University, Daqing, 163319 Heilongjiang People’s Republic of China

**Keywords:** Developmental biology, Genetics, Molecular biology

## Abstract

Calmodulin-like proteins (CML) are important calcium signal transduction proteins in plants. CML genes have been analyzed in several plants. However, little information on CML in *Phaseolus vulgare* is available. In this study, we identified 111 *PvCMLs* distributed on eleven chromosomes. Phylogenetic analysis classified them into seven subfamilies. *Cis*-acting element prediction showed that *PvCML* contained elements related to growth and development, response to abiotic stress and hormones. Moreover, the majority of *PvCML*s showed different expression patterns in most of the nine tissues and developmental stages which indicated the role of *PvCML* in the growth and development of common bean. Additionally, the common bean was treated with melatonin by seed soaking, and root transcriptome at the 5th day and qRT-PCR of different tissue at several stages were performed to reveal the response of *PvCML* to the hormone. Interestingly, 9 *PvCML* genes of subfamily VI were detected responsive to exogenous melatonin, and the expression dynamics of nine melatonin response *PvCML* genes after seed soaking with melatonin were revealed. Finally, the protein interaction network analysis of nine melatonin responsive PvCMLs was constructed. The systematic analysis of the *PvCML* gene family provides theoretical support for the further elucidation of their functions, and melatonin response molecular mechanism of the CML family in *P. vulgaris*.

## Introduction

Calcium (Ca^2+^) is a dynamic element and one of the most important second messengers in plant cell signal transduction^1^. Calcium signaling plays a fundamental role in cellular activities. Different Ca^2+^ sensing proteins can detect different Ca^2+^ signals of cytoplasmic free calcium^2^. One of the most important protein chelators for Ca^2+^ is the protein with the EF-Hand domain^3^. Four important protein families contain EF-hand domains, including CAMs (Calmodulins)^4^, CMLs (calmodulin-like proteins)^5^, CDPKs (Ca^2+^-dependent protein kinases)^6^, and CBLs (Calcineurin B-like proteins)^7^.

CMLs are novel plant-specific Ca^2+^ sensors with the plant-specific EF-Hand domain^8^. The structure and biochemical features of CML proteins indicate that they have similar properties to CAM^3^. However, there is one Dx_3_D motif in CML and four calcium-binding DxD motifs in CAM^9^. It is an important distinction between CML and CAM. By bioinformatic methods, *CML* genes have been identified and analyzed in many plants, such as 50 *CML* genes in *Arabidopsis thaliana*^5^, 32 *CML* genes in rice^10^, 52 *CML* genes in tomato^11^, 144 *CML* genes in soybean^12^, 46 *CML* genes in *Medicago truncatula*^13^, 62 *CML* genes in grapevine^14^, and 79 *CML* genes in *Brassica rapa* L.^15^. In general, closely related members of CML family have high sequence similarity, and motif distribution and gene structure were arranged similarly in the same subfamily. Moreover, *CML* genes have been shown to play important roles in plant growth and development, cell metabolism, innate immunity, abiotic and biological stress disease resistance^5,16–19^. For example, the *ShCML*44 gene of tomato involved in responses to various stress, and the overexpression of *ShCML*44 can improve the tolerance of plants to cold, drought, and salt stress^20^. *AtCML8* expression can be induced by salicylic acid or NaCl treatment^21^. The expression of *AtCML*9 was induced by abscisic acid and some abiotic stresses, which indicated that *AtCML*9 plays a role in salt tolerance by modulating ABA-mediated pathways^16^. *AtCML20* inhibited ABA-induced stomatal closure and drought tolerance^22^, whereas *AtCML*24 inhibited pollen tube growth, autophagy, abscisic acid response, and ion stress^23^. In addition, a large number of *cis*-elements related to abiotic stress and hormones were predicted in the promoter region of the *CML* gene. Furthermore, previous experiments reveal that these *CML* genes showed different expression profiles after stress and hormone treatment^20^.

Melatonin (MT) was discovered in the bovine pineal gland in 1958^24^. Plant melatonin appears to be a multi-regulatory molecule with a variety of hormone activities in plants, including the regulation of seed germination rate, plant growth, and development process^25,26^. *Phaseolus vulgaris* L. is an edible leguminous plant^27^. Due to its economic value, especially in developing countries, many researchers have focused on the yield increase of the common bean^28^. Exogenous application of melatonin can regulate the growth and development of the common bean and significantly improve root growth of the common bean under salt stress^29^. Moreover, previous studies revealed that exogenous melatonin was sensed by receptor CAND2/PMTR1, which activated downstream of Ca^2+^ signal transduction depending on calcium sensors, including CMLs (Cam-like proteins)^30^. Therefore, exogenous melatonin response in the common bean is closely related to intracellular calcium and CML. However, the features, expression patterns, and the response to melatonin of the *CML* family in common bean is unclear until now.

In this paper, the *CML* gene family of common beans was identified, and there were 111 *PvCML*s in *P. vulgaris*. Its gene structure and *cis*-acting element distribution were analyzed. Subsequently, we analyzed publicly available transcriptome databases and determined the spatial–temporal expression pattern of all *PvCML* genes in several tissues. Our root transcriptomic data further revealed specific melatonin responsive *PvCML*, which belonged to a CMLs subfamily VI. Spatial–temporal expression profiling in different tissues of nine specific melatonin responsive CMLs was verified using qRT-PCR. Meanwhile, we also analyzed the expression dynamics of nine melatonin response *PvCML* genes in the root at different time points after melatonin seed-soaking treatment using qRT-PCR. This work provides a foundation for further functional study of *CML* genes and the molecular mechanism of melatonin.

## Materials and methods

### Identification of *CML* in *P. vulgaris*

Protein sequences of *P. vulgaris* (v2.1) were downloaded from Phytozome database (v13) (https://phytozome.jgi.doe.gov)^31^. The reported CML protein sequences in *A*. *thaliana* and rice were downloaded from the TAIR database (https://www.arabidopsis.org)^32^ and TIGR database (http://rice.plantbiology.msu.edu)^33^, respectively. Then, 32 *OsCML* and 50 *AtCML* proteins were used as query sequences to perform BLASTP searching (E-value < 1e−5)^34^. To remove false *PvCML* genes, we screened the candidate *CML* genes according to the main character of conserved EF-hand domains^9^. Subsequently, InterPro 86.0 (http://www.ebi.ac.uk/interpro) and SMART 9.0 (http://smart.embl-heidelberg.de/) software was applied to verify the reliability of the EF-hand domain prediction. Additionally, a Dx_3_D motif was also checked to distinguish the CML family from the CAM family.

### Chromosomal location and physicochemical characterization of *PvCML* genes

The sequences of *PvCML* were used to retrieve their chromosomal locations in the *P*. *vulgaris* genome databases Phytozome 12. The software TBtools was used to analyze the chromosomal location. Each *PvCML* gene was named on the basis of its precise position on the chromosome.

The physicochemical properties such as amino acid number, isoelectric point (pI), molecular weight, and coefficient of protein were analyzed by Protparam tool of ExPASy (https://web.expasy.org/protparam). The Plant-mPLoc tool (http://www.csbio.sjtu.edu.cn/bioinf/plant-multi) was used to predict subcellular localization of CMLs in *P*. *vulgaris*.

### Phylogenetic analysis of CML

Multiple sequences alignment of 111 PvCML proteins were performed using Clustal W, and a phylogenetic tree was constructed by MEGA X with the Maximum-likelihood method, and Jones–Taylor–Thornton (JTT) + G model was set, and 1000 bootstrap replicates was performed.

### Analysis of conserved domain, gene structure and *cis*-acting element of *PvCML* genes

The exon–intron structure analysis of *PvCML* genes was conducted using the TBtools program with default parameters. The conserved motifs were analyzed with MEME (https://meme-suite.org). The 2.0 kb upstream sequences of *PvCML* genes were analyzed using the PlantCARE (http://bioinformatics.psb.ugent.be/webtools/plantcare/html) to identify the *cis*-acting elements in the promoter region of *PvCML*.

### Duplication events analysis of CML

The *PvCML* was mapped to the chromosomes according to the chromosomal locations provided by Ensembl plants database (https://plants.ensembl.org/index.html). The gene duplication events were analyzed by Multiple Collinearity Scan toolkit (MCScanX) with default parameters. We used TBtools with default parameters to calculate Ka, Ks, Ka/Ks.

### Tissue specific expression profile of *PvCML* gene family

RNA-seq data from 9 tissues or organs were retrieved from the phytozome database. The transcript data of the *PvCML* gene family were selected and sorted, then a heatmap was constructed by the TBtools software. Fragments per kilobase of exon per million fragments mapped (FPKM) value was transformed to log_2_ (value + 1)^35^.

### Gene expression analysis of *PvCML* gene response to melatonin treatments

Common bean varieties are provided by the National Coarse Cereals Engineering Research Center. In this study, *P. vulgaris L. *var. Jiyin 1 was analyzed and the formal identification of the plant materials was undertaken by Mr. M. Li. We got the permission to collect the plant samples and all methods were carried out in accordance with relevant guidelines and regulations. Our root transcriptome data of *P. vulgaris* after melatonin treatment at 5 day was analyzed as above, *PvCML* genes response to melatonin treatments were identified. The assembled gene dataset, deposited at the National Center for Biotechnology Information under the accession number PRJNA55837615 (http://www.ncbi.nlm.nih.gov/bioproject/).

To further study the melatonin effect on the expression of these *PvCML* genes, *P. vulgaris* L. var. Jiyin 1 (JY) was treated with 100 µmol/L melatonin by seeds soaking, which had the most obvious effect on the growth of common bean, and distilled water was used as a control (H_2_O). Then, JY was cultured in petri dish (ϕ12 × 1.5 cm) for 7 days at the temperature 25 °C in dark, and radicle were sampled at different stages (3 d, 5 d, 7 d). Meanwhile, some JY treated by melatonin was planted in soil and cultured in greenhouse with 18 h/8 h light at 25 °C. The leaf, stem, hypocotyl, and root of JY were sampled at 10 d. All samples were immediately frozen in liquid nitrogen and stored at − 80 °C for further experiments.

Total RNA of all samples (hypocotyls, stems, leaves, and roots) was extracted by TRIzol reagent (BIOMARS, Beijing, China). RNA was subsequently reverse-transcripted to cDNA using a SuperMix (Innovagene, Beijing). For qRT-PCR primers of ninmelatonin responsive genes, all 111 *PvCML* nucleotide sequences were aligned, and the primers were designed in the region of difference. Moreover, these specific primers were further confirmed by primer-blast analysis. The qRT-PCR primers were listed in Supplementary Table S1. The normal PCR was performed by these primers, and the PCR products were detected on agarose gel electrophoresis to make sure of the purity of amplification. Then, qRT-PCR was conducted using the CFX96 qPCR system (Bio-Rad). The qRT-PCR protocol was as follows: 95 °C for 3 min; 40 cycles of 95 °C for 15 s and 60 °C for 30 s. Three biological replicates and three technical replicates were performed. *Actin* (KF569629) was used as an internal reference gene, and the relative expression level of *PvCML* genes were calculated using 2^−ΔΔCt^^36^.

### Protein interaction analysis and visualization

The protein interaction network of PvCML proteins which responded to exogenous melatonin was analyzed using the STRING website (https://www.string-db.org), and the protein interaction network was visualized with Cytoscape software^37^. Moreover, annotation of these proteins was performed in the KEGG website (https://www.kegg.jp/)^38^.

## Results

### Identification and characterization of CML family members in *P*. *vulgaris*

**Figure 1:**
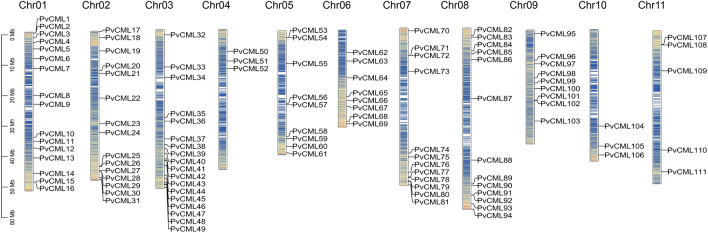
Distributions of *PvCML* genes on the *P. vulgaris* chromosomes. Chromosome numbers are located at the top of each bar. Sizes of chromosomes are represented by the vertical scale. The scale is in megabases (Mb).

In total, 124 CML protein sequences with EF-hand domains were identified in *P. vulgaris* using BLASTp, and 111 genes (*PvCML*1-111) were confirmed and classified in the CML family by InterPro and SMART analysis. These *PvCML* genes sequences were further used to retrieve the chromosomal locations of *PvCML* genes. The results showed that *PvCML* genes were distributed on all chromosomes of *P. vulgaris*. Specifically, 16 *PvCMLs*, 15 *PvCMLs* and 18 *PvCMLs* were found on chromosomes 1, 2 and 3, respectively. Three *PvCML*s were distributed on chromosomes 4 and 10; chromosomes 5 and 9 contained nine *PvCML*s; chromosome 6, 7, 8, and 11 contained eight, twelve, thirteen and five *PvCML*s, respectively (Fig. 1).

Physicochemical properties analysis showed that the predicted molecular weight of *PvCML* genes ranged from 9240.24 Da (*PvCML* 24) to 89,386.22 Da (*PvCML* 1), and the number of amino acids of the PvCMLs ranged from 80 AA (*PvCML* 24) to 789 AA (*PvCML* 1). The predicted pI varied from 3.90 (*PvCML* 73) to 9.79 (*PvCML* 97). The subcellular localization results showed that 54 *PvCML*s were located on the cell membrane, 29 *PvCML*s on the nucleus, 3 *PvCML*s on the cell membrane and nucleus, and the remaining *PvCML*s on the cytoplasm, vacuole, and chloroplast (Supplementary Table S2).

### Evaluation of gene structures and conserved motifs of the *CML*s in *P. vulgaris*

**Figure 2:**
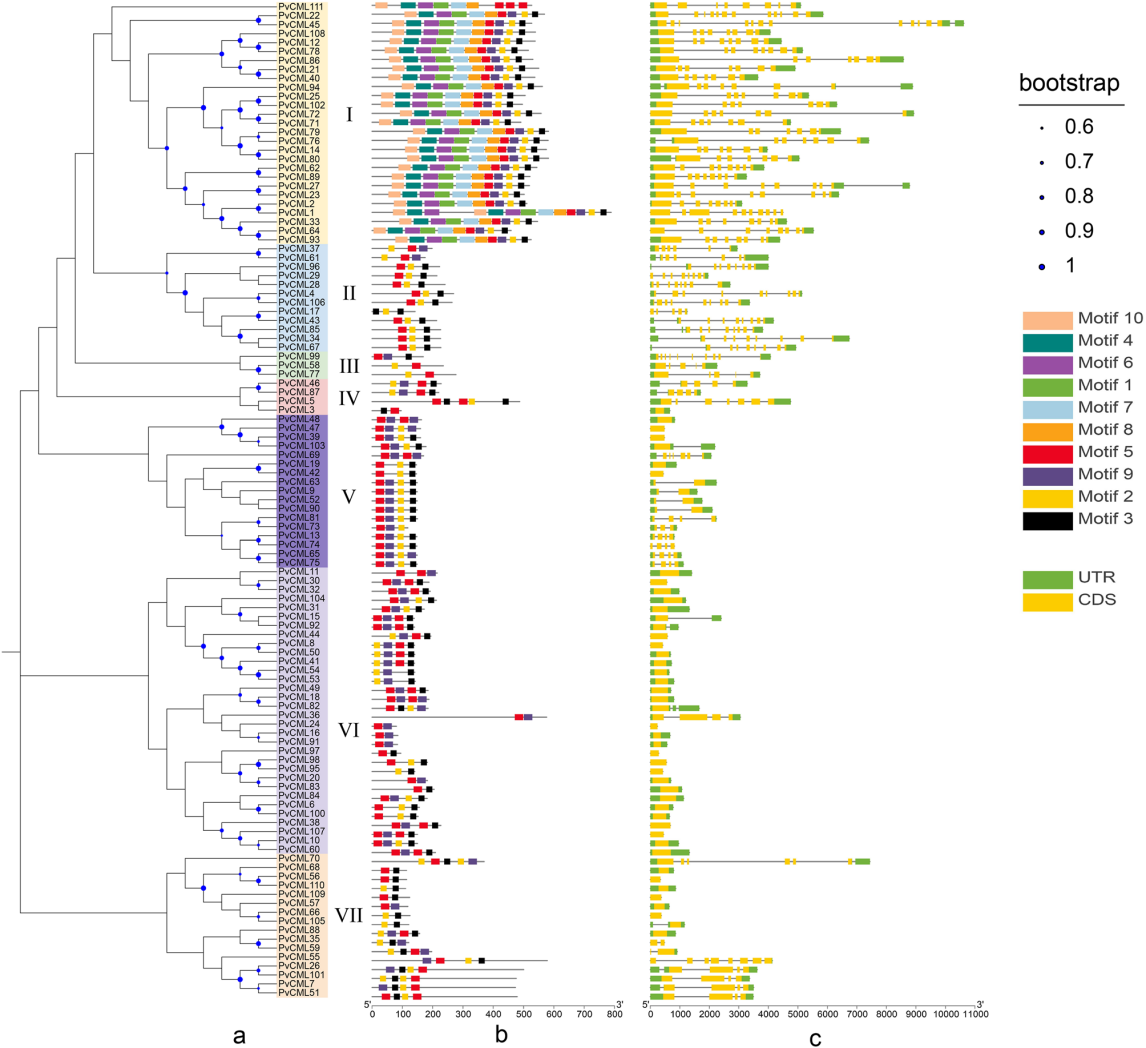
Phylogenetic relationships, gene structure, and motif compositions of *PvCML* genes. (**a**) Phylogenetic tree of *PvCMLs*. The phylogenetic tree was constructed with the maximum-likelihood method of MEGA X (1000 bootstrap replicates). The size of the blue circle represented bootstrap value. (**b**) Motif structure of *PvCMLs*. Different motifs were represented by different colors. Motif lengths were presented proportionally. (**c**) The gene structure of *PvCMLs*. The yellow box represented exon, and the green box represented the untranslated region. Intron represents by solid line.

A phylogenetic tree was constructed based on the alignment of PvCMLs full-length amino acid sequences by MEGA software to demonstrate the structural classification of the PvCMLs. PvCMLs could be divided into seven subfamilies (I–VII) (Fig. 2a). Furthermore, conserved motifs analysis of these proteins showed that all 111 PvCMLs contained at least one EF-hands domain with D-x_3_-D structure. Ten different motifs were identified and their distribution were shown in Fig. 2b. The conserved sequences of ten motifs were listed in Supplementary Fig. S1. Each PvCML subfamily had a unique motif distribution model. Motif 1, 4, 6, 7, 8, and 10 were specifically presented in the subfamily I members. These motifs are protein kinase domains which are usually associated with ATP binding and protein kinase activity. Other family members (II-VII) were different in order or quantity of motif 2, 3, 5, and 9 (Fig. 2b). These four motifs were EF-hand domains associated with calcium-binding. Meanwhile, according to the motif sequence (Fig. S1), motifs 2, 3, 5, and 9 are EF-hand domains containing Dx_3_D structures. Dx_3_D was in the first place of EF-hand domain in 111 PvCML, which was consistent with the characteristics of CML proteins.

To gain information about the conservation and difference of *PvCMLs* genes, we analyzed the exon–intron organization of *PvCML*s genes. The results showed that the number of exons in *PvCMLs* ranged from 1 to 12, and forty-four *PvCML*s (39.63%) contained only one exon. There were twenty-six *PvCML*s (23.42%) with more than 8 exons. Most *PvCML*s (53.15%) contained 2 to 7 exons (Fig. 2c). The subfamily I–V members and some members of subfamily VII contained multiple exons. The majority of subfamily VI members contained one exon. Above all, the phylogenetic tree and gene structure analysis demonstrated that the structural difference between *PvCMLs* may be related to its gene function within this family.

### Prediction of *cis*-elements in the promoter sequences of *PvCMLs* genes

**Figure 3:**
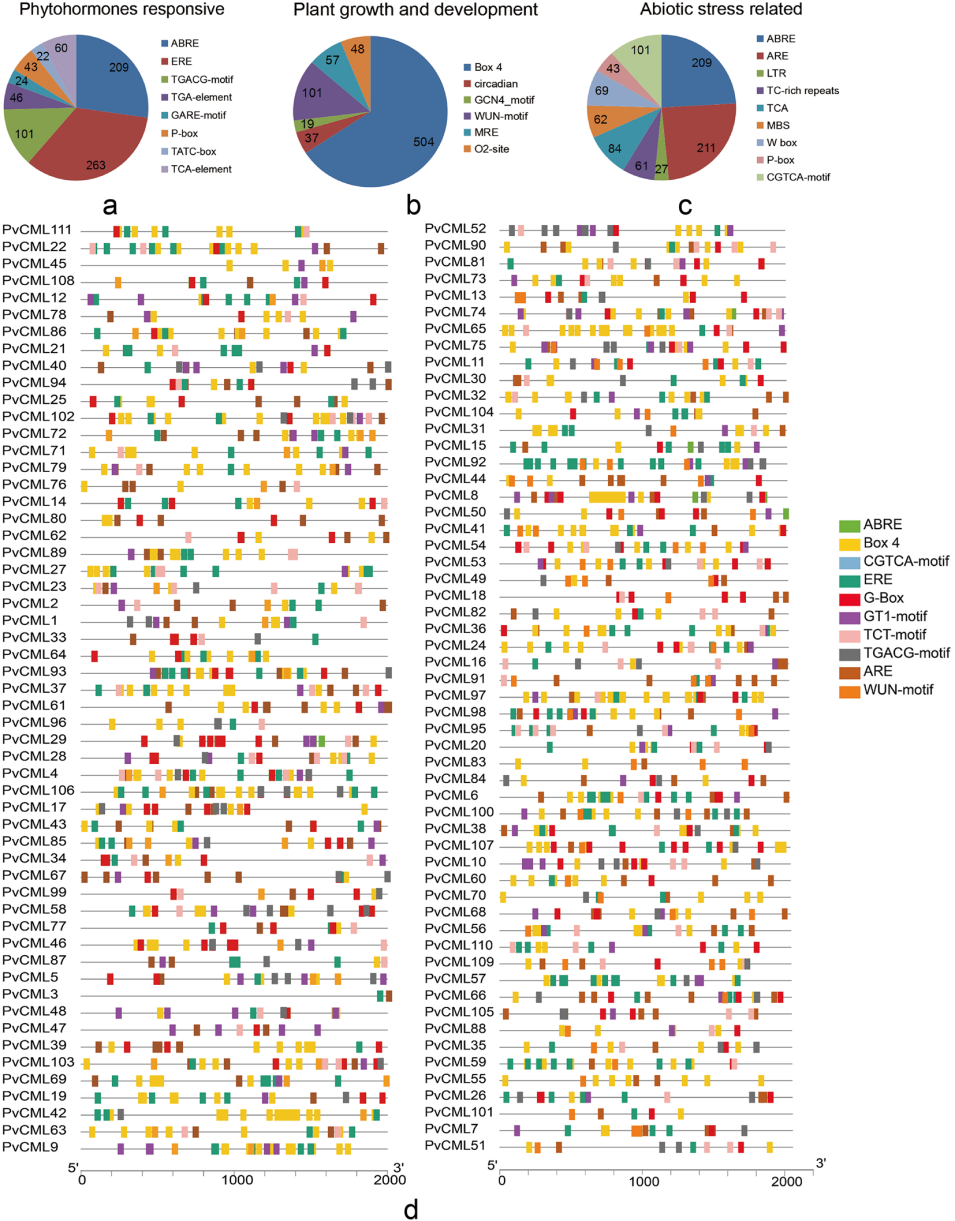
*Cis*-elements in the promoter regions of *PvCMLs.* (**a**) The number distribution of phytohormones responsive element, (**b**) plant growth and development related element, (**c**) abiotic stress response element and (**d**) distribution of top 10 *cis*-regulatory elements in each *PvCMLs* gene. Element is indicated by different colors in the grid.

To explore the transcriptional regulation of *PvCMLs* gene, *cis*-elements in *PvCMLs* promoter regions (the 2000 bp upstream region from transcriptional start site) were predicted using Plant CARE. A total of 99 *cis*-regulatory elements were identified. Partial *cis*-acting elements of *PvCML* were further classified into plant hormone response element, growth and development response element, and abiotic stress response element. Phytohormone (ABA, MeJA, GA, and SA) responsive elements included TATC-box, ABRE, TCA-element, CGTCA-motif, P-box, GARE-motif, TGACG-motif, etc. (Fig. 3a). *Cis*-elements involved in plant growth and development were widely distributed in all genes (Fig. 3b). Additionally, four types of abiotic stresses (drought, low-temperature, anaerobic, and light) related elements, such as ABRE, ARE, LTR, GT1-motif, MBS, etc. were identified (Fig. 3c). The top ten *cis*-regulatory elements, with the exception of CAAT and TATA-box, were visualized, including 504 Box 4, 263 ERE, 241 G-box, 211 ARE, 208 ABRE, 142 GT1-motif, 106 TCT motif, 101 CGTCA-motif, 101 WUN-motif (Fig. 3d). These results indicated that *PvCML*s genes might play critical roles not only in various plant developmental events, but also in phytohormone and abiotic responses in *P. vulgaris.*

### Phylogenetic analysis of PvCML proteins

**Figure 4:**
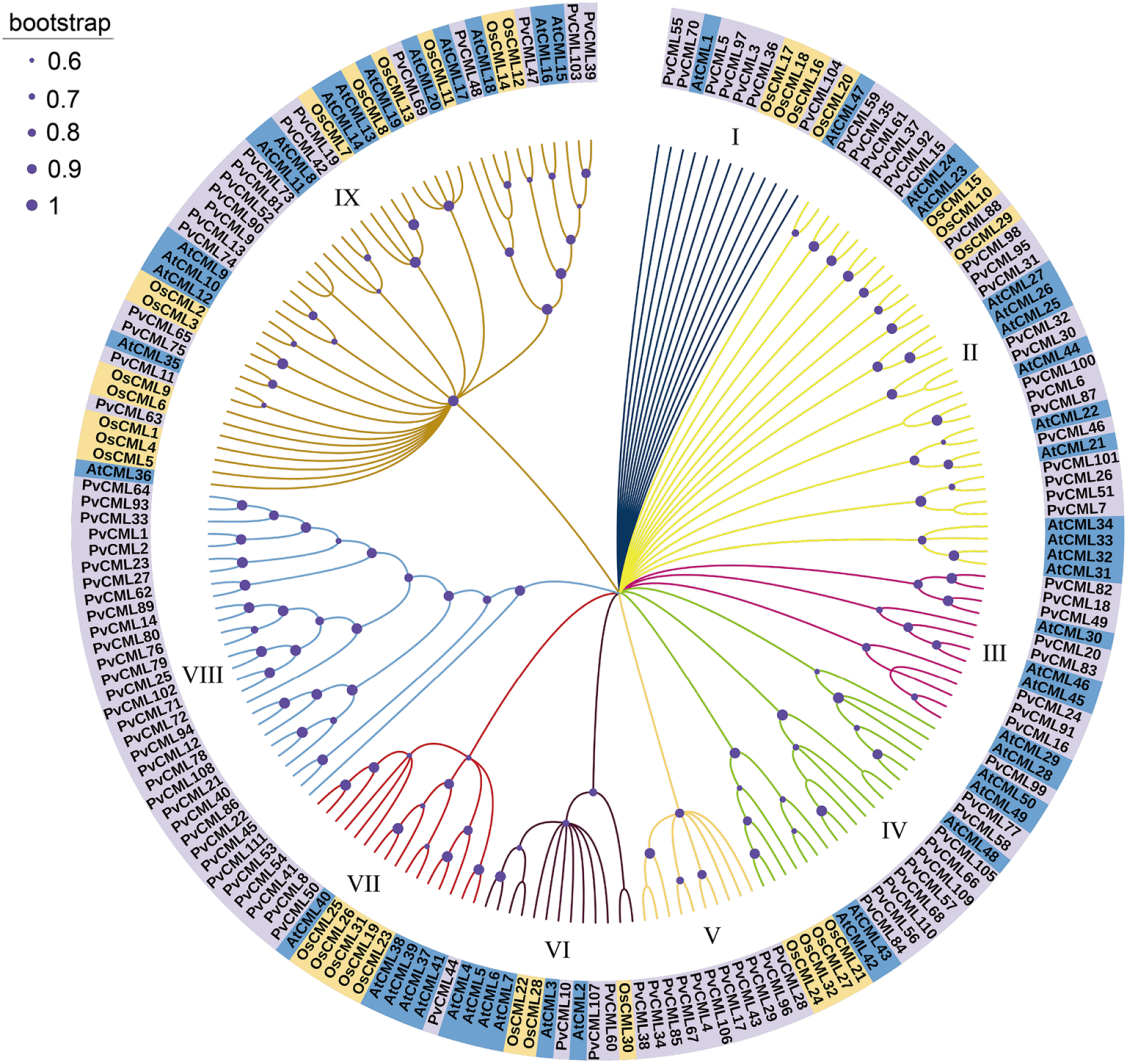
Maximum likelihood tree of CML proteins from *P. Vulgaris*, *O. sativa* and *A. thaliana*. The *CML* proteins in the branches from different species were indicated by different color: purple, *P. Vulgaris*; yellow, *O. sativa*; blue, *A. thaliana*. Different colors of branches represented different subfamilies. The size of the blue circle represented the bootstrap value.

To evaluate the evolutionary relationships of the CMLs among common bean (*P*. *vulgaris*), rice (*Oryza sativa *L.) and *A*. *thaliana*, multiple sequence alignment was performed using the CML amino acid sequences. A comprehensive phylogenetic tree was constructed with 193 CML protein sequences, including 111 sequences from common bean (PvCMLs), 32 from rice (OsCMLs), and 50 from Arabidopsis (AtCML). These CML genes were classified into nine subgroups (Group I–Group IX) (Fig. 4). Group I included 13 CML members (2 AtCML, 4 OsCMLs and 7 PvCMLs); Group II included 35 CML members (12 AtCMLs, 3 OsCMLs and 20 PvCMLs); Group III contained 13 CML members (5 AtCMLs and 8 PvCMLs); Group IV included 20 CML members (5 AtCMLs, 4 OsCMLs and 11 PvCMLs); Group V and VIII subgroups only contained PvCML members, PvCML 28/96/29/43/17/106/4/67/85/34/38 and PvCML 64/93/33/1/2/23/27/62/89/14/80/76/79/25/102/71/72/94/12/78/108/21/40/86/22/45/111 formed a single V and VIII subgroups in the phylogenic tree; Group VI contained 13 CML members (6 AtCMLs, 3 OsCMLs and 4 PvCMLs); Group IX included 46 CML members (15 AtCMLs, 13 OsCMLs and 18 PvCMLs).

### Gene duplication and synteny analysis

**Figure 5:**
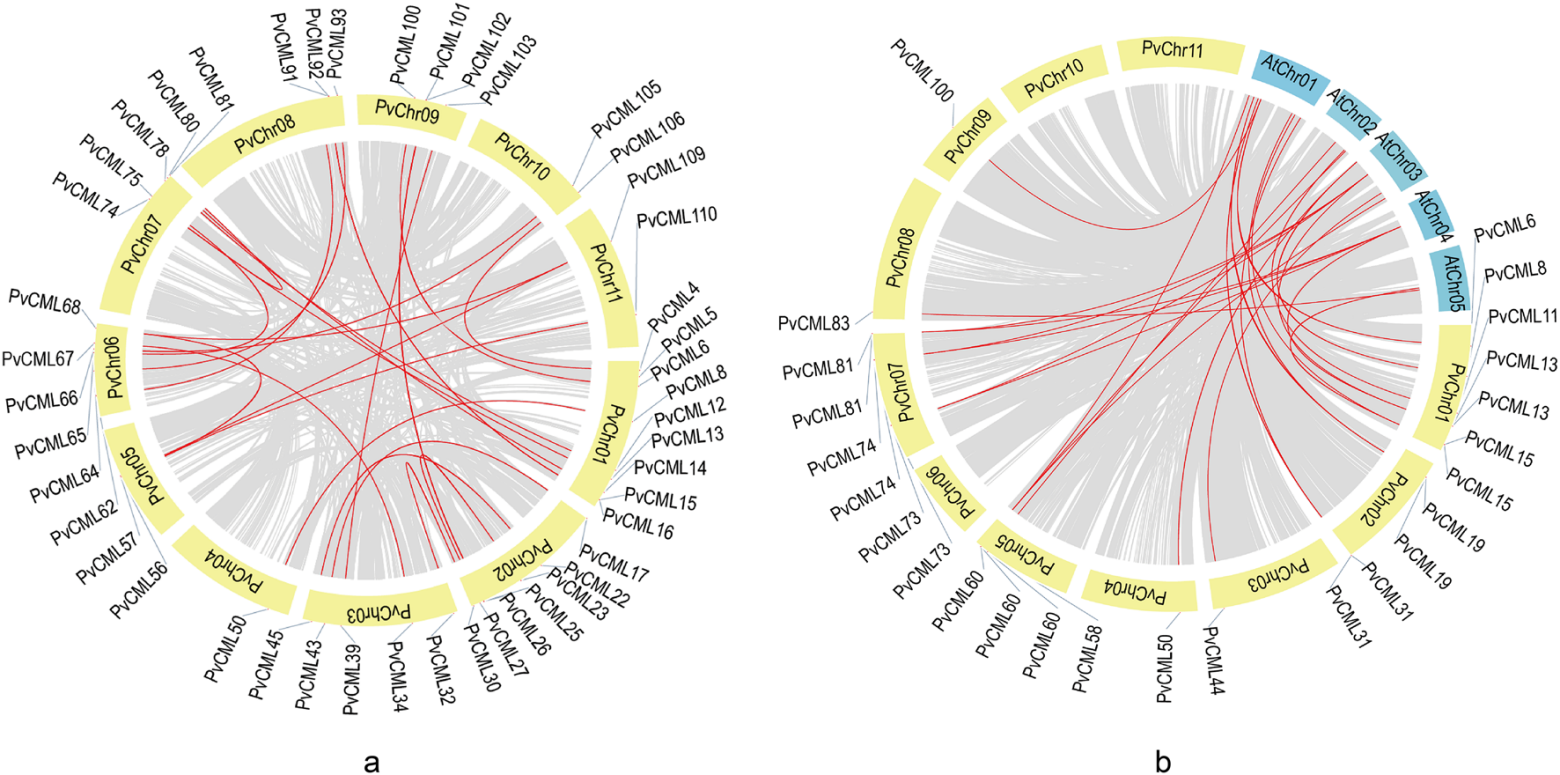
Duplication events of *CML* genes. The dispersed and segmental duplication events of *PvCML* genes in *P. vulgaris* (**a**) and the synteny relationships of *PvCML* genes between *P. vulgaris* and *Arabidopsis* (**b**). Red lines indicated segmental duplication of *PvCML* genes.

To clarify the *PvCML* gene duplication events in common bean, the segmental duplication events in *PvCML* gene family were investigated. As shown in Fig. 5a, twenty-one gene duplication events formed by genes from different chromosomes. Chromosome 4 contains a duplicated segment, and chromosome 1, 2, 3, 6, and 7 each contain more than five *PvCML* duplicated segments. Other chromosomes (4, 5, 8, 9, 10, and 11) each contain no more than five duplicated segments. There are two pairs of duplicated segments on the same chromosome. To elucidate the role of selection pressure in evolution, the Ka, Ks, and Ka/Ks values of duplicated genes of the *PvCML* family were calculated using the TBtools software. Except for no value of two gene pairs (*PvCML* 39/*PvCML* 103 and *PvCML* 56/*PvCML* 68), the value of Ka/Ks of other duplicated *PvCML* gene pairs were obtained (Supplementary Table S4). The results showed that the mean Ka/Ks of dispersed duplication (35%) was 0.1834, while the mean Ka/Ks of the segmental duplication (65%) was 0.1534. The Ka/Ks values of all duplication events were < 1 which indicated that the driving force for *PvCML* family evolution was the purification selection.

In addition, the synteny relationship of *CML*s between *P. vulgaris* and *A. Thaliana* was analysed. Twenty-five pairs of homologous *CML*s were identified between *P. vulgaris* and *A. thaliana* (Fig. 5b). Seven genes (*PvCML* 13/15/19/31/73/74/81) had two homologs in *Arabidopsis*, while one gene (*PvCML* 60) had three homologs in *Arabidopsis* (Supplementary Table S4).

### Expression profile analysis of *PvCML* genes in different tissues and melatonin treatment

**Figure 6:**
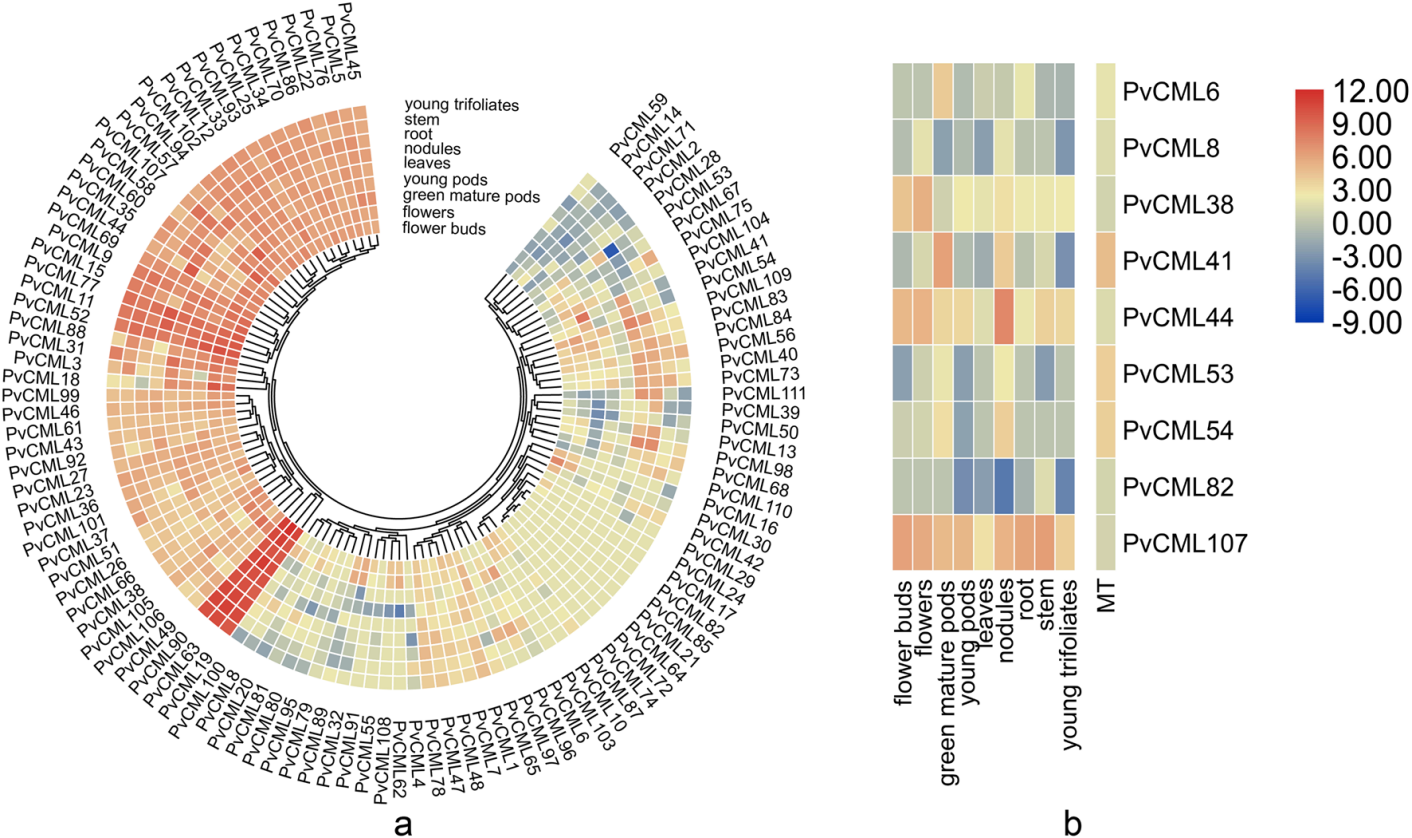
Transcriptome analysis of *PvCML* gene under different tissues and melatonin treatment. (**a**) Expression pattern of *PvCML* genes in different tissues based on the transcriptome data. (**b**) The expression profiles of *PvCML* genes in root after melatonin treatment. Transcript levels were depicted by different colors on the scale. Blue and red represented low and high expression levels, respectively.

Global gene expression data of. *P. vulgaris* are publicly available, including expression profiles of *PvCML*s gene at different developmental stages. To explore the possible functions of the *PvCML*s, the public transcript data of the *PvCML*s genes in 9 different tissues and organs, including leaves, stem, nodule, root, flowers, flower buds, young pod, green mature pods, young trifoliates, were analyzed. The majority of *PvCML*s showed different expression patterns in most of 9 tissues and developmental stages (Fig. 6a). Three *PvCML* genes (*PvCML* 19/63/90) were highly expressed in all tissues, whereas five *PvCML* genes (*PvCML* 59/14/71/2/28) were expressed at low expression levels in most stages. Some *PvCML*s genes showed a tissue specific expression pattern. *PvCML* 17 and *PvCML* 24 were expressed only in flower buds. *PvCML* 53 was expressed at low expression levels in the root. *PvCML* 10 and PvCML 103 were highly expressed in flowers and flower buds. In addition, some *PvCML* genes showed similar expression patterns, implying that they may have similar functions in plant growth and development.

In view of the presence of hormone-related *cis* elements in *PvCML* gene promoters, we further analyzed the effects of melatonin on the expression of *PvCML* genes. According to the previous experiment, we treated seeds of common bean with 100 μM melatonin by seed soaking, and the root transcriptome on the fifth day was sequenced.

Our transcriptome data from melatonin-treated roots revealed that nine *PvCML* genes (*PvCML* 6/8/38/41/44/53/54/82/107) in the exogenous melatonin treatment group displayed significantly higher expression levels than that of the control group, and the nine genes all belonged to the subfamily VI (Fig. 6b). This result indicated these genes in group VI plays a more important role in plant response to melatonin. Meanwhile, eight of the nine *PvCML* transcription patterns were identified from public transcriptome data except *PvCML* 82. *PvCML* 6 and *PvCML* 41 were highly expressed in green mature pods, *PvCML* 8 in flower tissues and roots, *PvCML* 38 in flower-related tissues, *PvCML* 44 in nodules, *PvCML* 53 and *PvCML* 54 in green mature pods and roots; Transcript level of *PvCML* 107 in all tissues were consistent.

### qRT-PCR analysis of nine melatonin responsive *PvCML* genes

In order to verify the reliability of transcriptomic data, we used qRT-PCR to detect the expression levels of nine melatonin responsive *PvCML* genes at four developmental stages (3 d root, 5 d root, 7 d root, and 10 d root) after melatonin treatment and in different tissues (10 d root, 10 d stem, 10 d hypocotyl, and 10 d leave).

**Figure 7:**
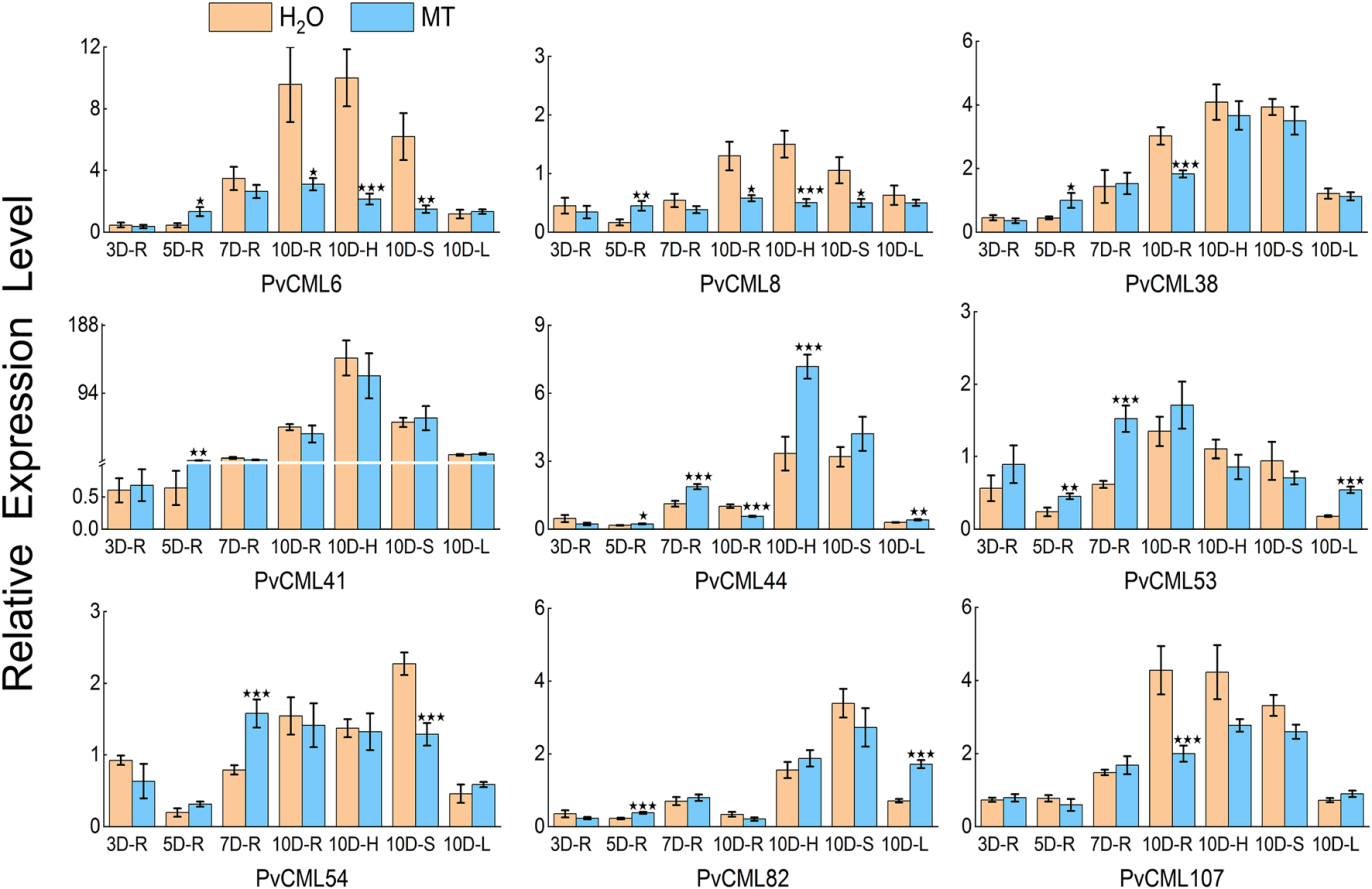
The qRT-PCR expression patterns of *CML* genes in *P. vulgaris.* The reactions were normalized using the *actin* reference gene. The standard deviations were represented by the error bars from three independent technical replicates. 3 Day-Root (3D-R), 5 Day-Root (5D-R), 7 Day-Root (7D-R), 10 Day-Root (10D-R), 10 Day-Hypocotyl (1D-H), 10 Day-Stems (1D-S) and 10 Day-Leaf (1D-L). The expression levels of each gene were expressed as a ratio relative to that of 3D-R (H_2_O). *t*-test was used to analyze the mean expression levels of three replicates in melatonin treatment (MT) with control treatment (H_2_O). Star(s) above the bars indicated significant differences among the treatments. A star represented significant level (*p* < 0.05), two stars represented significant level (*p* < 0.01), and three stars represented significant level (*p* < 0.001).

In the control group (H_2_O), the expression changes dynamics of nine genes in roots at different time points can be divided into three types. *PvCML* 6/38/107 displayed a consistently increasing trend. *PvCML* 8/41/53/54 decreased at first, then showed an increasing trend across the subsequent two stages. However, *PvCML* 44/82 decreased in the initial two stages, then increased obviously at 7 d, and decreased at 10 d again (Fig. 7). In the treatment group, *PvCML* 6/38 displayed a consistent increasing trend along the four stages in the root, and *PvCML* 41/107 displayed an increasing trend at most stages except for a decrease at 7 d and 5 d, respectively. The expressions of *PvCML* 44/53/54/82 were slightly upregulated in the initial three stages and then decreased at 10 d. *PvCML* 8 kept a stable expression trend along the four stages (Fig. 7). In addition, the expression of nine *PvCML* genes were detected in different tissues in the melatonin treatment and control group. The control group showed higher content in roots and hypocotyls, for example, six genes (*PvCML* 6/8/38/41/44/82) were dominantly expressed in hypocotyls, while two genes (*PvCML* 53/107) were dominantly expressed in roots. *PvCML* 54 was dominantly expressed in stems. Expression of these nine genes in leaves was at a low transcript level (Fig. 7).

After melatonin treatment, five genes (*PvCML* 6/8/38/44/54/82) of treatment group showed a downregulation trend compared with the control group on the third day of root development. Three genes (*PvCML* 41/53/107) in treatment group were up-regulated. On the fifth day of root development, melatonin treatment significantly upregulated eight genes except *PvCML* 107. The results were primarily consistent with the transcriptome data. On the seventh day of root development, seven genes (*PvCML* 6/38/44/53/54/82/107) were up-regulated compared with the control group. On the tenth day of root development, all genes except *PvCML* 53 were down-regulated by melatonin. On the tenth day of hypocotyl development, genes except *PvCML* 44/82 were down-regulated by melatonin treatment. On the tenth day of stem tissue, genes except *PvCML* 41/44 were down-regulated by melatonin treatment. On the tenth day of leaf tissue, genes except *PvCML* 8/38/41 were up-regulated by melatonin treatment (Fig. 7).

### Interaction network of PvCML proteins

**Figure 8:**
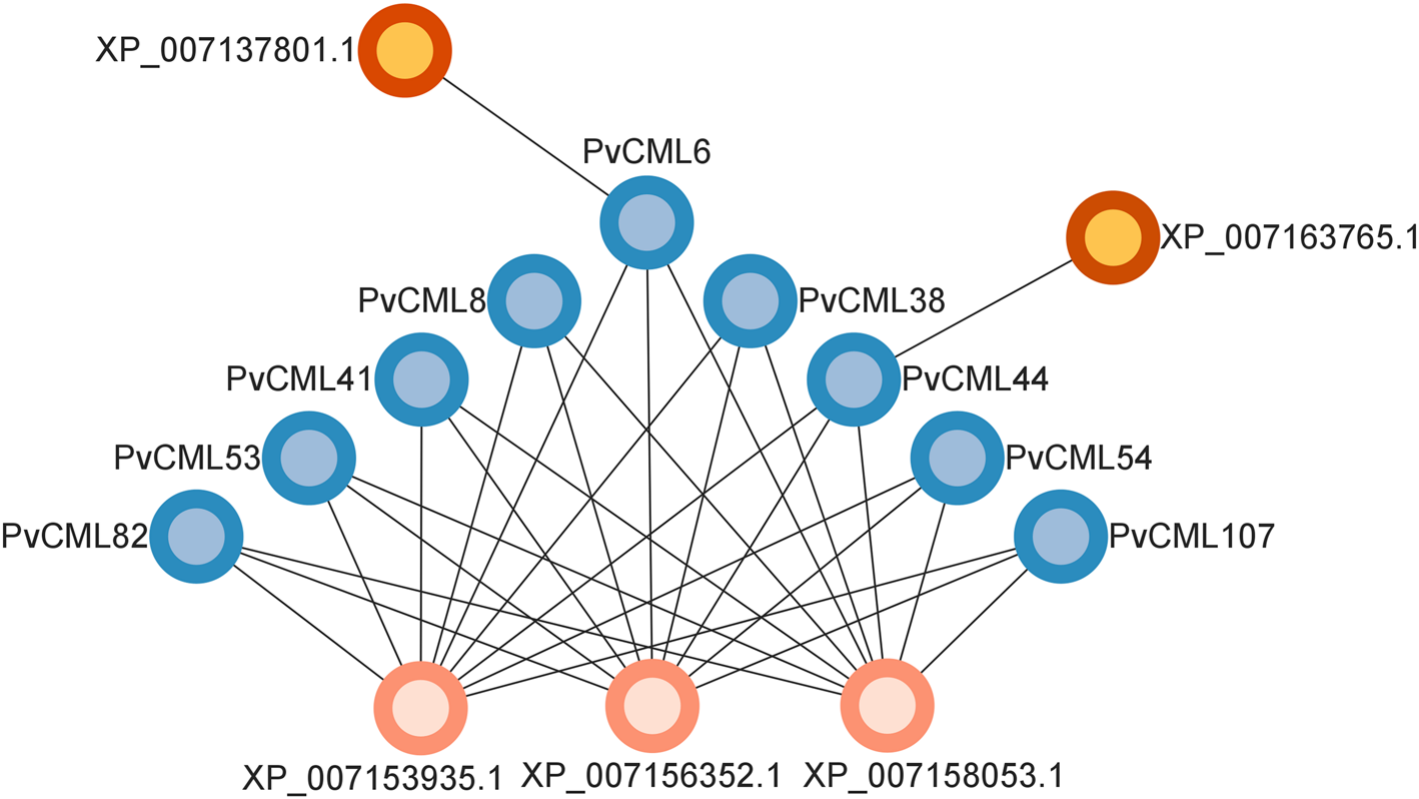
Interaction network of the nine PvCML proteins.

STRING database was used to construct an interaction network of nine melatonin responsive PvCML proteins. KEGG results indicated that *PvCML* 6/8/38/41/44/53/54/82/107 were mainly involved in plant–pathogen interaction pathways. Only three genes were found to interact with *PvCML* 6/8/38/41/44/53/54/82/107 genes (Fig. 8). Among them, XP _007153935.1 contains motif CH (actin binding domain), ARM, and IQ (calmodulin binding motif). XP_007156352.1 and XP_007158053.1 both contain motif MyTH4 (plant driver protein), B41 (plasma membrane binding domain), and KISc (driver protein). By GO analysis, it was found that XP_007153935.1, XP_007158053.1, and XP_007156352.1 genes are jointly involved in ATP binding, ATP-dependent microtubule motor activity, microtubule binding, oxidoreductase activity, and calmodulin binding pathway. XP_007137801 and XP_007163765 interacted with PvCML 6 and PvCML 44, respectively (Fig. 8). So far, XP_007137801 and XP_007163765 were uncharacterized proteins.

## Discussion

Under biotic or abiotic stress, plant cells will produce a specific pattern of intracellular calcium flux, which will trigger the signal cascade and eventually affect the calcium concentration in cell^39,40^. As a kind of primary calcium sensors, plant *CML* proteins play key roles in cellular signaling networks by regulating various targets^41,42^. With the accumulation of various plant genome sequences, *CML gene family has* become a typical feature of plant genome^43^. However, the characteristics of CML gene in common bean have not been systematically studied. In this study, 111 *PvCML* genes were identified from the common bean genome by bioinformatic methods. The amount of *PvCML* in common bean was equivalent to that in other leguminous crops, such as soybeans^12^. Compared with *A*. *thaliana*^44^, *O. sativa*^10^ and Chinese cabbage^15^, common beans have more *CML* genes. The number of *CML* gene family members varies significantly among plant genomes, and it is independent of genome size. The variation of *CML* gene number among different species may be caused by the natural variation of different species and their adaptation to the complex growth environment.

Gene length, amino acid number, and pI of each *PvCML* were different. However, compared with *CML* gene families in other dicotyledons, the average number of amino acids of *PvCML* (282) was similar to that of *MdCML* (231). The mean pI of *PvCML* (5.17) and *MdCML* (5.22) were essentially the same (Supplementary Table S5). Some *PvCML* genes that shared close phylogenetic relationships displayed similar EF-hand distributions and intron–exon structures, which indicate that these genes may have similar biological functions and expression characteristics. The function of CML members depends on the number of EF-Hand motifs involved in Ca^2+^ binding properties^45^. The number of EF-Hand motifs varies among plant species. For example, *AtCML* usually contains 2–6 EF-Hand motifs^5^. Whereas *MtCML* contains 1–4 EF-Hand motifs^13^. In common beans, 2–4 conserved EF-Hand motifs were found in *PvCML*. Some motifs, such as motifs 2, 3, 5, and 9, calcium-binding EF-Hand domains, were conserved in all subfamilies of PvCML, and these motifs were related to the basic function of PvCML. Motifs 1, 4, 6, 7, 8, and 10, protein kinases, were unique in the subfamily I, which may affect the ATP binding and protein kinase activity of the subfamily I PvCML. And each PvCML contains a Dx_3_D motif. In addition, intron–exon structure analysis in *M*. *truncatula* and *Brassica rapa* showed that 78% of *MtCML* genes and 76.92% of *BraCML* genes did not contain introns^15^. Our Intron–exon structure analysis showed that 36.04% of *PvCML* genes did not contain introns. Compared with *MtCML* and *BraCML*, *PvCML* contains fewer genes without introns. It is thought that the fewer introns in a gene, the faster a plant responds to environmental changes^46,47^. In order to further understand *PvCML*, a phylogenetic tree was constructed to explore the evolutionary relationship of *PvCML*. In fact, 23 *PvCML* duplication pairs (20.72%) in 111 *PvCMLs* were identified in the common bean genome which suggested that some *CML* genes may be generated by segmental duplication events. Meanwhile, 25 *PvCML* were homologous with *AtCMLs*. Similarly, 20 pairs of homologous *CML* genes were found in *M. truncatula* and *Arabidopsis*^13^. At the same time, previous study suggested that whole-genome duplication (WGD) of legumes plays an important role in shaping the genome^46^. Therefore, WGD and segmental duplication may be involved in the evolution of *PvCML* gene.

In this study, we found that at least one stress response *cis*-element, growth and development related element, and hormone response element were detected in each *PvCML* gene. The results indicated that *PvCML* plays an important role in plant growth and development, stress response, and hormone response. For example, *AtCML* 44 is a homologous gene of *PvCML* 6. *AtCML* 44 is up-regulated under drought and salt stress. *OsCML* 31 is a homologous gene of *PvCML* 41. *OsCML* 31 is strongly induced under drought stress, and its overexpression can enhance plant drought tolerance^48^. *AtCML* 41 is a homologous gene of *PvCML* 44. *AtCML* 41 has been proved to be a stress response gene and plays an inhibitory role in the basic defense response of plants^49,50^. *Cis*-acting element analysis also showed that *PvCML 6, PvCML 41,* and *PvCML* 44 contain MBS (MYB binding site drought response element). *AtCML 42* is a homologous of *PvCML 84 and PvCML 98. AtCML 42* has been shown to affect plant response by changing plant hormone signals. *Cis*-acting element analysis showed that *PvCML 84* and *PvCML 98* contained TGACG *cis*-acting elements in response to hormones*.*

In addition, the tissue specific expression pattern of *PvCML* gene is thought to be related to its potential biological function. Transcriptome data analysis showed that the expression of the same subfamily members in the same tissue was not consistent. *PvCML* gene was highly expressed in different tissues, which was basically consistent with previous reports of *M*. *sativa* and Arabidopsis. For example, previous experiments showed that *AtCML* 3 was highly expressed in flower organs^51^. *AtCML 3* homologous *PvCML 107* also showed a higher expression in floral apparatus in transcriptome data.

Transcriptomic analysis of common bean treated with melatonin showed that nine *PvCML* genes were differentially expressed in the root of common bean. Subsequently, these nine genes were verified by qRT-PCR, and the results were consistent with the transcriptome data. In normal treatment or melatonin treatment, the expression of nine *PvCML*s in roots was up-regulated or fluctuated with time, and reached the highest on the 7th or 10th day. In previous studies, *CML*s in other plants were also significantly affected by hormone treatment, for example, MeJA induced high expression of *AtCML* 39 with more than 100 times^52^. *CMLs* expressions were different at different development stages in common bean. qRT-PCR results showed that the expression levels of nine *PvCML* genes in leaves were lower than that in other tissues. In addition, exogenous supplementation of low concentration melatonin can promote the growth of hypocotyls, stems, and roots of plants^53–55^. Our study proved that exogenous melatonin affected the expression of *PvCML* 6, *PvCML* 8, and *PvCML* 44 in hypocotyl, but increased the expression of *PvCML* 44/53/82 in leaves. The expression of *PvCML* 6/8/54 were downregulated in stems by melatonin. Taken together, differential expression of the *PvCML* gene at various time points and different tissues of the common bean suggested its response to melatonin treatment. More experiments need to be performed to demonstrate how melatonin regulates *PvCML*.

Interaction network analysis of genes contributes to the understanding of their functions. Hence, protein interaction net of nine melatonin responsive *CML* was analyzed. Three proteins were identified (XP_007153935.1, XP_007158053.1, and XP_007156352.1), which were involved in ATP binding, ATP-dependent microtubule movement, and microtubule binding and calmodulin binding pathways, respectively. The interaction between *CML*s and these genes requires further study.

## Supplementary Information

Supplementary Figure S1.

Supplementary Table S1.

Supplementary Table S2.

Supplementary Table S3.

Supplementary Table S4.

Supplementary Table S5.

## Data Availability

All data generated or analysed during this study are included in this published article and its supplementary information files.
