# Supplementary figures and images for: Genome-wide analysis of the CML gene family and its response to melatonin in common bean (Phaseolus vulgaris L.)
Source: Sci Rep. 2023 Jan 21;13:1196. doi: 10.1038/s41598-023-28445-y (PMC9867747; doi:10.1038/s41598-023-28445-y)

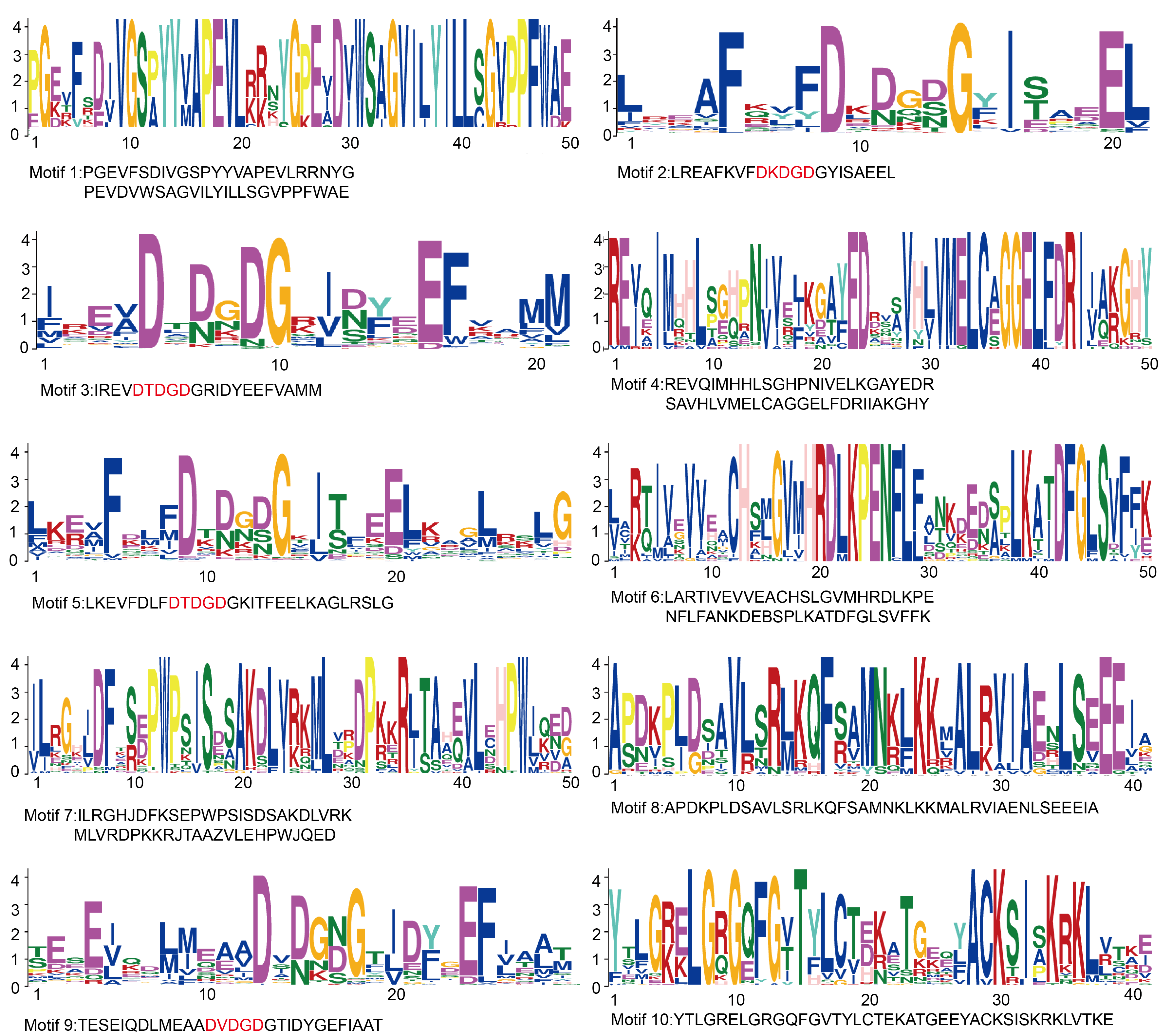

Supplement: Supplementary file 1 — Supplementary Figure S1. [file 41598_2023_28445_MOESM1_ESM.png]
